# Microhydration of Deprotonated Nucleobases

**DOI:** 10.1007/s13361-016-1411-3

**Published:** 2016-05-13

**Authors:** Henryk Wincel

**Affiliations:** Institute of Physical Chemistry, Polish Academy of Sciences, 01-224 Warsaw, Poland

**Keywords:** Hydration energies, Deprotonated nucleobases, High-pressure mass spectrometry

## Abstract

Hydration reactions of deprotonated nucleobases (uracil, thymine, 5-fluorouracil,2-thiouracil, cytosine, adenine, and hypoxanthine) produced by electrospray have been experimentally studied in the gas phase at 10 mbar using a pulsed ion-beam high-pressure mass spectrometer. The thermochemical data, *ΔH*^*o*^, *ΔS*^*o*^, and *ΔG*^*o*^, for the monohydrated systems were determined. The hydration enthalpies were found to be similar for all studied systems and varied between 39.4 and 44.8 kJ/mol. A linear correlation was found between water binding energies in the hydrated complexes and the corresponding acidities of the most acidic site of nucleobases. The structural and energetic aspects of the precursors for the hydrated complexes are discussed in conjunction with available literature data.

**Graphical Abstract:**
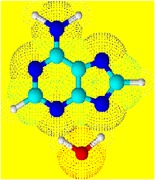
ᅟ

ᅟ

## Introduction

Hydrogen bonding plays a central role in biological structures and function, including protein and nucleic acid folding, molecular recognition, signal transduction, and enzymatic catalysis [1]. Hydrogen bonds in DNA and the interaction between two complementary nucleobases, which are held together by NH–O and NH–N hydrogen bonds, are dependent on the intrinsic basicity of the acceptor atoms as well as on the acidity of the donor groups [2, 3]. The strength of these bonds is related to the p*K*a values of the components [4]. The hydrogen bonding between the nucleobases (NB) in DNA and RNA duplexes is very important for a greater understanding of their structure and function in vivo [5].

When ionizing radiation interacts with living organisms, the low-energy electrons (<15 eV) efficiently damage DNA by inducing single- and double-strand breaks [6]. These alterations are initiated by dissociative electron attachment (DEA) with the initial capture of an electron leading to a temporary negative ion, which may decompose by spontaneous ejection of the electron or by dissociation into neutral and anionic fragments [6, 7]. Gas- phase studies have shown that the most abundant fragment anions formed via the DEA process of uracil [8], thymine [9, 10], cytosine [11], 2-thiouracil [12], adenine [13], and hypoxanthine [14] are the deprotonated nucleobases [NB-H]^–^. The formation of these anions is energetically driven by the electron affinity of the [NB-H]^•^ radicals, which lie in the range between 3 and 4.5 eV [9, 11, 15, 16].

A large amount of computational [17–42] and experimental [20, 27, 31, 32, 36–39] investigations has been carried out in order to determine the acidities of nucleobases. Several of these studies were focused on the examination of the properties of deprotonated uracil and its derivatives [17, 18, 20–22, 24, 26–31, 35–37, 39, 40, 42], cytosine [23, 28, 34, 37, 39], adenine and its derivatives [19, 32, 33, 37, 41], hypoxanthine [38], and guanine [41] in the context of the mechanism of action of the enzymes, which recognize damaged bases and remove them from DNA. For example, the mechanism for uracil excision from the genome by the enzyme uracil DNA glycosylase (UDG) involves nucleophilic attack by some form of activated water of the *N*-glycosidic bond connecting the nucleobase to the sugar and formation of N1^–^ deprotonated uracil as the leaving group [43, 44].

Although it is essential to characterize the properties of deprotonated forms of isolated nucleobases, it is equally important to examine their properties in environments that mimic some of the aspects of the biological world. Water is the natural medium of biological systems, and for that reason our investigations are focused on the hydration of different ionic forms of nucleobases. In our previous studies, we investigated the thermochemical properties for the gas-phase hydration of protonated nucleobases and protonated nucleosides [45], sodiated and potassiated nucleobases [46], and protonated and sodiated thiouracils [47].

**Scheme 1:**
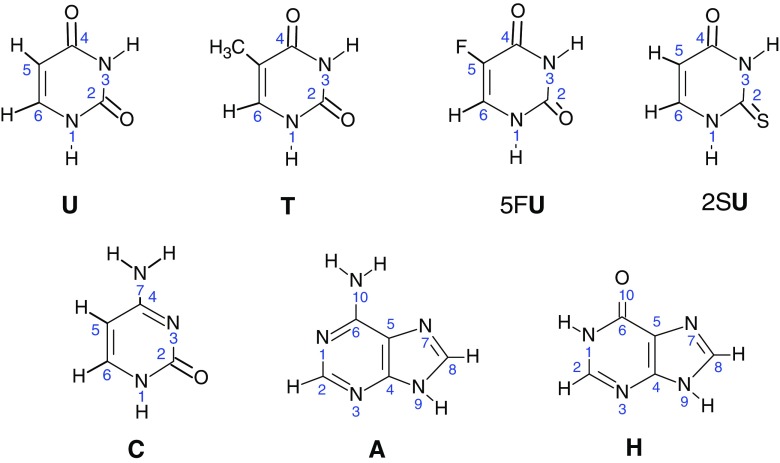
Structures and atom numbering of the systems considered in the present study

In this paper, we present the experimental investigations of the interactions of one molecule of water with deprotonated uracil [**U**-H]^–^, thymine [**T**-H]^–^, 5-fluorouracil [5F**U**-H]^–^, 2-thiouracil [2S**U**-H]^–^, cytosine [**C**-H]^–^, adenine [**A**-H]^–^, and hypoxanthine [**H**-H]. Schemetic structures and atom labeling of neutral nucleobases are shown in Scheme 1.

The five nucleobases (**U**, **T**, **C**, **A**, and **G**) are directly involved in the formation and the stability of the well-known double helix structure of DNA and RNA. We could not conduct measurements with **G** (guanine) as it is sparingly soluble in the electrospraying solution. **H** is a mutagenic purine base that most commonly arises from the oxidative deamination of **A**, and is associated with carcinogenesis and cell death [38]. Modified nucleobases, 5-F**U** and 2S**U**, are important and interesting compounds because of their biological and pharmacological properties. 5-F**U** is widely used in the treatment of a range of cancers, including colorectal and breast cancers, and cancers of aerodigestive tract [48, 49]. 2-S**U** has found medical applications as antithyroid and anticancer drugs [50–52].

Several theoretical studies on the interaction of deprotonated nucleobases with water have been performed. Kryachko et al. [22] estimated the binding energies of water molecule with the N3^–^ anions of 2-S**U**, 4-S**U**, and 2,4-dS**U**. Wetmore and co-workers [29, 30] computationally investigated the binding energies of neutral and the N1^–^ anionic uracil and its derivatives with small molecules (NH_3_, H_2_O, or HF) at the O_2_(N3), O_4_(N3), and O_4_(C5) binding positions. Their results showed that the binding strengths are relatively independent of the substituent. Furthermore, they reveal decrease in the deprotonation energy at N1 by about 20 kJ/mol with one associated water to uracil [29]. Computational studies by Bachrach and Dzierlenga [42] have indicated that the difference (54.4 kJ/mol) in deprotonation energy between the N1 and N3 sites of uracil decreases with each added water up to four. At this point, the energy difference has been halved, but addition of a fifth or sixth water has little effect on the energy difference. The Wetmore group [34] carried out density functional theory studies of the complexes between NH_3_, H_2_O, or HF molecules and four main binding sites in neutral and N1 deprotonated cytosine. They found that the trends in the effects of hydrogen bonds on the N1 acidity are similar for all pyrimidines. To the best of our knowledge, no experimental results on the gas-phase hydration of deprotonated nucleobases have been reported.

## Experimental

The experiments were performed with a high-pressure mass spectrometer using a pulsed ion-beam ESI ion source, which has been described in detail elsewhere [53]. Briefly, the reactant ions were produced by electrospraying water/acetonitrile (20%:80%) solutions containing ~2.0 mM nucleobase to which a few drops of ammonium hydroxide were added. The pH value of solution measured with Schott CG 837 (Mainz, Germany) instrument was ~10.5. Each solution was supplied to a silica capillary (15 μm i.d., 150 μm o.d) by a syringe pump at a rate of 0.8 μL/min, and a negative voltage was held at approximately 4 kV.

The clustered ions were desolvated by a dry nitrogen gas counter current and in a heated (~80°C) pressure-reducing capillary through which they were introduced into the fore-chamber, and then deflected toward a 3-mm orifice in the interface plate leading to the reaction chamber (RC). Ions drifting across the RC toward the exit slit under the influence of a weak electric field (2 V/cm at 10 mbar) were hydrated and reached equilibrium prior to being sampled to the mass analysis section of the mass spectrometer. Ion detection was provided by a channeltron equipped with a conversion dynode. The output pulses of the multiplier were counted using a multichannel scaler with dwell-time per channel of 1 μs. Mass spectra were registered with continuous ion sampling, while for equilibrium determination the ion beam was injected into the RC in a pulsing mode by applying short pulses (–52 V, 200 μs) to the deflection electrode. The latter mode of operation allows for measurements of the arrival time distribution (ATD) of the ions across the RC.

The reagent gas mixture consisting of pure N_2_ as the carrier gas at about 10 mbar and a known partial pressure of water vapor (0.1–0.25 mbar) was supplied to the RC via the heated reactant gas inlet (RGI) at a flow rate of ~100 mL/min. The pressure was measured with an MKS capacitance manometer attached near the inlet of the RGI. The amount of water introduced into the N_2_ gas flow was kept constant throughout the temperature-dependent measurements of the equilibrium constants. Water concentrations were controlled continuously with a calibrated temperature and humidity transmitter (Delta OHM, Type DO 9861T; Casselle di Selazzano, Italy). The RC temperature was monitored by an iron-constantan thermocouple, which was embedded close to the ion exit slit; the temperature can be varied from ambient to ~300°C by electrical heaters.

The chemicals, N_2_ (Polish product, 99.999%) and the nucleobase samples: uracil, thymine, cytosine, adenine, and hypoxanthine obtained from Aldrich Chemical Co. (Steinheim, Germany), 2-thiouracil from Alfa Aesar GmbH & Co. KG (Karlsruhe, Germany), and 5-fluorouracil from abcr GmbH & Co. KG (Karlsruhe, Germany) were used without further purification. The water was deionized with a Millipore purifier, type Elix 5 (Vienna, Austria).

**Figure 1:**
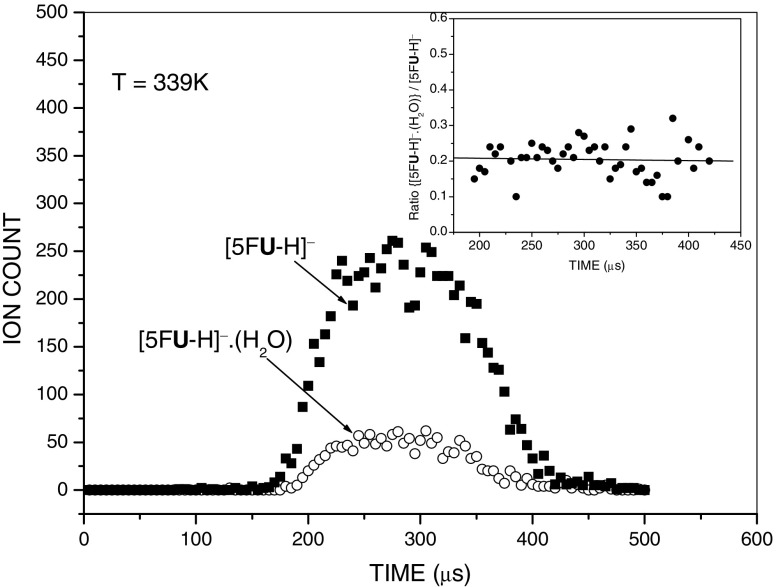
Arrival time distributions of the reactant, [5F**U-**H]^−^, and product, [5F**U-**H]^−^⋅(H_2_O), ions. The inset shows the ratio of ion intensities,{[5F**U-**H]^−^⋅(H_2_O)}**/** [5F**U-**H]^−^ as a function of ion residence time

The gas-phase hydration energies of deprotonated nucleobases were determined by measurement of the equilibria described by the general reaction (1)

1 $$ {\left[\mathrm{N}\mathrm{B}\hbox{-} \mathrm{H}\right]}^{\hbox{--}}\cdotp\;{{\left({\mathrm{H}}_2\mathrm{O}\right)}_{n-}}_1 + {\mathrm{H}}_2\mathrm{O}\ \leftrightarrow\ {\left[\mathrm{N}\mathrm{B}\hbox{-} \mathrm{H}\right]}^{\hbox{--}}\cdotp\;{\left({\mathrm{H}}_2\mathrm{O}\right)}_n $$

for which the thermodynamic equilibrium constant is

2 $$ {K_{n-}}_{1,n} = \left({I}_n\cdotp {P}_o/{I_{n-}}_1\cdotp P\right) $$

where *I*_*n*_ and *I*_*n-*1_ are recorded ATD peak areas of [NB-H]^–^٠(H_2_O)_*n*_ and [NB-H]^–^٠(H_2_O)_*n*__-1_ respectively, and *P* is the known partial pressure of water (in mbar). The standard pressure *P*_*o*_ is 1000 mbar. Equilibrium attainment in the RC was verified by comparing the ATDs of the reactant and product ions, and testing that the *I*_*n*_**/***I*_*n-*1_ ratio was independent of ion residence time. A typical example of such tests is shown in Figure 1 for the (0,1) hydration step of [5F**U**-H]^–^. The inset of the figure shows that within the error limits and the limits of statistical noise, the ratio {[5F**U**-H]^–^٠(H_2_O)}/[5F**U**-H]^–^ remains essentially constant, suggesting the attainment of equilibrium for the system.

Measuring *K*_*n-*1,*n*_ as a function of temperature *T* and using the thermodynamic relationships (3) and (4)

3 $$ \ln\;{K}_{n-1,n} = \left(\varDelta {S^o}_n/R\ \right) - \left(\varDelta {H^o}_n/RT\ \right) $$

4 $$ \varDelta {G^o}_n=\varDelta {H^o}_n-T\varDelta {S^o}_n $$

the values for the enthalpy, *ΔH*^*o*^_*n*_ , entropy, *ΔS*^*o*^_*n*_*,* and free energy, *ΔG*^*o*^_*n*_ , of Reaction 1 were obtained. The weighted least-squares fitting procedure was used to obtain the slopes and intercepts of each line. The slopes determine the enthalpy change (*ΔH*^*o*^_*n*_) and the intercepts yield the corresponding *ΔS*^*o*^_*n*_ value. The uncertainty corresponds to the standard deviation of the linear least-squires fit.

During these experiments, we determined thermochemical data for the hydration Reaction 5 to support the validity of the present results and provide bases for comparison with the data obtained in previous studies [54] (see Table 1).

**Table 1:** Experimental Enthalpies, Entropies, and Free Energy Values^a^ for the Hydration of Deprotonated Nucleobases^a^

Ion	*–ΔH* ^*o*^_*n*_ (kJ/mol)	*–ΔS* ^*o*^_*n*_ (J/mol K)	*–ΔG* ^*o*^_*n*_ (kJ/mol)^b^	Acidity (kJ/mol)	
[2S**U** – H]¯	39.7(2); *46.5* ^c^	67.4(10)	19.7(6)	1365.3^d^ (N1)	1411.6^d^ (N3)
[5F**U** – H]¯	40.6(2); *41.9* ^e^	65.3(5)	21.1(4)	1376.5^f^ (N1)	1435.1^f^ (N3)
[**U** – H]¯	43.0(2); *42.6* ^e^	64.4(7)	24.0(4)	1393.3^f,g,h^ (N1)	1451.8^f,g^ (N3)
[**T** – H]¯	43.5(2); *43.3* ^e^	71.1(8)	22.5(4)	1401.6^i^ (N1)	1447.7^i^ (N3)
[**C** – H]¯	44.8(2)	70.3(6)	23.8(4)	1422.6^f^ (N1)	1447.7^f^ (N7)
[**A** – H]¯	42.7(2)	68.2(5)	22.7(4)	1393.3^f,j^(N9)	1472.7^f,j^ (N10)
[**H** – H]¯	42.2(2)	63.0(6)	23.4(4)	1389.1^k^(N9)	1539.7^k^
**J**¯	42.0(2)	68.2(8)	21.7(4)		
	42.3^l^	66.1l^l^	22.6^l^		

Standard pressure is 1000 mbar.

^a^ Uncertainties in parentheses.

^b^ *–ΔG* ^*o*^_*n*_ at 298 K.

^c^ Calculated water binding energy for the N3 anionic complex of [2S**U** – H]¯• (H_2_O), configuration **1c**, Ref. [22].

^d^ Ref. [35].

^e^ Calculated water binding energy for the [NB – H]¯• (H_2_O) complex deprotonated at N1, configuration **1b**, Ref. [30].

^f^ Ref. [37].

^g^ Ref. [20].

^h^ Ref. [31].

^i^ Ref. [39].

^j^ Ref. [32].

^k^ Ref. [38].

^l^ Ref. [54].

5 $$ \mathrm{J}\bar{\mkern6mu} + {\mathrm{H}}_2\mathrm{O}\ \leftrightarrow\ {\mathrm{J}}^{\hbox{--}}\left({\mathrm{H}}_2\mathrm{O}\right) $$

## Results and Discussion

**Figure 2:**
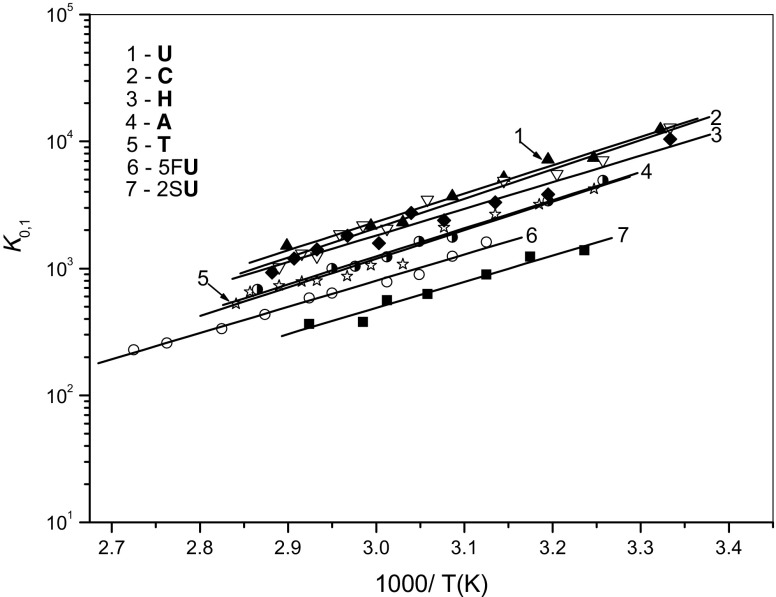
van’t Hoff plots of equilibrium constants for the gas-phase reactions: [**NB**-H]^−^ + H_2_O ↔ [**NB**-H]^−^ ⋅(H_2_O). Compounds **NB** are given in the figure

The van’t Hoff plots for the temperature studies of the hydration reactions of [NB-H]^–^ are shown in Figure 2 and the results are summarized in Table 1, along with related literature data. The results show that the hydration enthalpies, *ΔH*^*o*^, for all anions are essentially the same, and the small differences can be attributed to the correlation with the gas-phase acidities of nucleobases. The data will be presented elsewhere. In this work, the term “gas-phase acidity” is used to refer to the enthalpy change, *ΔH*^*o*^_*ac*_, associated with deprotonation. Table 1 shows the gas-phase acidities of the most acidic and the less acidic site of nucleobases. For all these nucleobases, more than one site in the molecule can be deprotonated. Similarly to the neutral nucleobases, their deprotonated forms can exist in several tautomeric structures, and the measured hydration enthalpy changes for [NB-H]^–^ may represent an average over several contributing structures. The formation of [NB-H]^–^ by ESI could occur from different locations. The anions produced from aqueous solution may be different from those formed in the gas-phase region, in which changes can occur either in the transition of the ion from the charged droplet to the gas phase or in the gas phase due to ion-molecule reactions [55], where catalyzed isomerization can occur in the presence of neutral nucleobase [20]. The possible anionic structures of [NB-H]^–^ created by ESI that might be involved in the hydration equilibrium 1 are characterized in the following discussion.

### Uracil and Its Derivatives

**Scheme 2:**
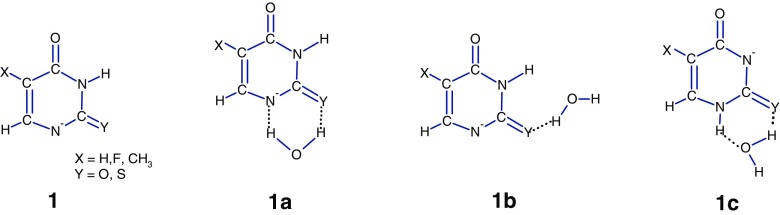
Structures of deprotonated uracil and its derivatives, and their complexes with water molecule

For uracil and its derivatives, the possible deprotonation sites are N1 and N3. In the gas phase, N1 is more acidic than N3, by about 45–60 kJ/mol (see Table 1), while in aqueous solution the N1 and N3 acidities of uracil are indistinguishable, and the N1^–^ monoanion is in equilibrium with that of N3^–^ in ca. 1:1 ratio [43]. A similar proportion also holds for the mixtures of the monoanions N1^–^ and N3^–^ in aqueous medium of thymine [56] and 2-thiouracil [57]. For 5-fluorouracil, the spectral data [58] show the predominance of N3^–^ in the N1^–^ and N3^–^ monoanionic mixture in aqueous solution. However, in alkaline aqueous solution, the situation can be different. Theoretical studies [59] show that in alkaline aqueous media, the deprotonation at N1, with equilibrium constant, *K*_eq_^(N1)^, should be the dominant path of uracil ionization. This result is supported by the reaction field calculations with the isodensity polarizable continuum (IPC) model, with the equilibrium constant ratio, *K*_eq_^(N1)^/ *K*_eq_^(N3)^ = 5 × 10^4^. In the case of 5F**U**, the N1^–^/ N3^–^ anion fraction ratio in aqueous alkaline solution was found to be 0.61 [60]. The N3^–^ anion, if formed in aqueous solution, in the gas phase can isomerize to N1^–^ in the presence of neutral nucleobase [20]. According to the in vacuo ab initio calculations, the N1^–^ anion of [**U**-H]^–^ is more stable than N3^–^ by 58.5 kJ/mol [59]; for [5F**U**-H]^–^ this difference is 49.9 kJ/mol [60]. The energy barrier (185.4 kJ/mol) calculated [61] for the uracil N1^–^ —> N3^–^ conversion is too high to be overcome at thermal energies in our instrument. Therefore, it is reasonable to assume that the N1^–^ would be the predominant form of the [NB-H]^–^ anions of uracil and its derivatives (structure 1 in Scheme 2) formed by ESI in the present study and these species are the most favorable precursors for hydrated complexes. Calculations [42] for the uracil N1^–^ predict that the most stable complex with water, **1a**, is formed when water is attached to the anion in a bidentate fashion between the deprotonated N1 and the adjacent carbonyl oxygen. Configuration **1b** and the complexes with water binding at the O4(C5) and O4(N3) positions in uracil (not shown in Scheme 2), are significantly (at least 12.6 kJ/mol) higher in energy than **1a** [42] and would be expected to be minor in abundance under the present experiments. It is very likely that the **1a** and **1b** structures are also formed from the hydrated structure **1** of [2S**U**-H]^–^, [5F**U**-H]^–^, and [**T** H]^–^.

As can be seen in Table 1, for the [**U**-H]^–^, [**T**-H]^–^, and [5F**U-**H]^–^ anions, the measured *ΔH*^*o*^ values are very close to the calculated [30] binding strengths between water and the N1^–^ anions in the O2(N3)–H_2_O complex, **1b**, with water bound to the carbonyl oxygen adjacent to N1^–^. In the case of structure **1c**, the computed [22] binding energy of water (46.5 kJ/mol) to the N3^–^ anion of [2S**U**-H]^–^ is significantly higher than the experimental hydration enthalpy value (39.7 kJ/mol, Table 1). This comparison supports that the N1^–^ anions of uracil and its derivatives are the dominant precursors for the hydrated complexes of [NB-H]^–^ observed under the present experiments.

### Cytosine

**Scheme 3:**
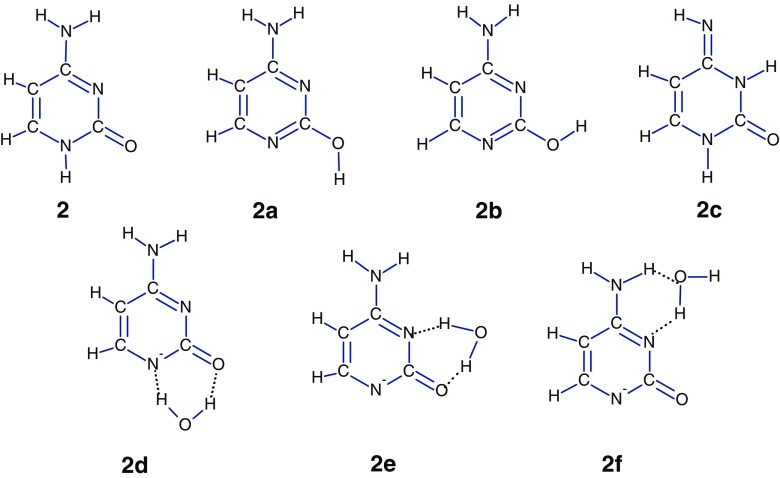
Structures of four tautomers of cytosine and their deprotonated complexes with water

According to the calculations [39], the canonical tautomer of cytosine, **2**, is the most stable and the three other most stable tautomers are higher in energy by 7.1 (**2a**), 10.5 (**2b**), and 9.2 kJ/mol (**2c**). The next most stable tautomer is predicted to be lying 16.7 kJ/mol higher in energy than **2** (Scheme 3).

As it has been shown [39] that the cytosine formed by electrospray of a methanol aqueous solution adopts predominantly the **2** form, where the most acidic site is N1. Thus, it might be expected that the N1^–^ anion of the tautomer **2** should be the dominant precursor of the [**C**-H]^–^ ٠(H_2_O) complex formed in the present experiments. The measured hydration energy for this complex (44.8 ± 2 kJ/mol, Table 1), is significantly lower than the water binding strengths calculated for the **2e** (57.1 kJ/mol) and **2f** (51.1 kJ/mol) complexes [34].These results imply that the **2d** complex dominates in the equilibrium reaction 1.

### Adenine

**Scheme 4:**
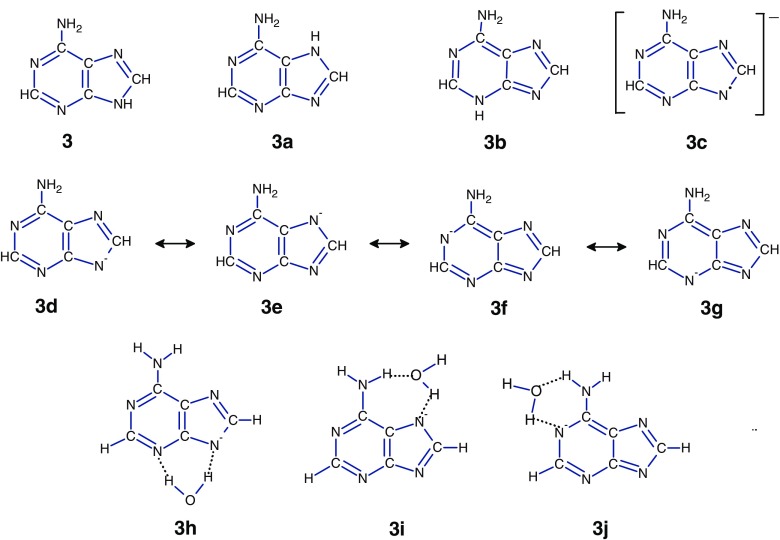
The three most stable tautomers of adenine (**3**, **3a**, and **3b**) and the resonance structures for deprotonated adenine (**3d**, **3e**, **3f**, and **3 g**), and their hydrated complexes (**3 g**, **3 h**, and **3i**)

In the gas phase, the canonical tautomer of adenine, **3**, is the most stable and predominant species. The next two tautomers, **3a** and **3b**, are higher in energy by ~34 kJ/mol [41, 62], Scheme 4.

Tautomerization **3** —> **3a** and **3** —> **3b** is predicted [63] to occur with a very large activation barrier (250–293 kJ/mol), indicating that the processes may not occur in the gas phase. In water, however, the energy difference between the canonical and these two tautomers is reduced to 4.7 kJ/mol (**3a**) and 18.0 kJ/mol (**3b**) [64]. The experimental measurements [65–67] and calculations [68] show that only the **3** and **3a** tautomers might be present in an aqueous solutions, and their population ratio, **3/3a**, was estimated to be in the range of 3.6–4.9 at 293 K.

In our experiments, the formation of [**A**-H]^–^ by ESI can occur from different locations of the parent molecule. In aqueous solution, these anions may result from the dominant tautomer **3** with possibly up to 20% of the **3a** tautomer. In the atmospheric pressure region, the ion formation predominantly from **3** may be expected. Therefore, it is very likely that the **3c** anion (Scheme 4) formed from a mixture of **3** and **3a** should be the precursor for the hydrated complexes.

The negative Mulliken charges predicted by theoretical studies [69] for the N atoms of the adenine N9^–^ are equal to 0.25e (N1), 0.25e (N3), 0.23e (N9), 0.23e (N7), and 0.20e (N10). These results suggest that the negative charge in **3c** is uniformly distributed, and a possibility exists that the resonance structures of this anion, **3d**, **3e**, **3f**, and **3 g**, can interact with the water molecule leading to the hydrated complexes **3 h**, **3i**, and **3j** (Scheme 4). It is also possible that we have a mixture of these complexes, and the hydration energies measured for these systems represent an average of their contribution. However, a comparison of the calculated [33] gas-phase acidities for the adenine N9H (334.8 kJ/mol), N7H (326.7 kJ/mol), N3H (326.7 kJ/mol), and N1H (316.3 kJ/mol) with the measured [32] acidity of the most acidic site N9H (333 ± 2 kJ/mol) may imply the predominant formation of the **3 h** complex.

### Hypoxanthine

**Scheme 5:**
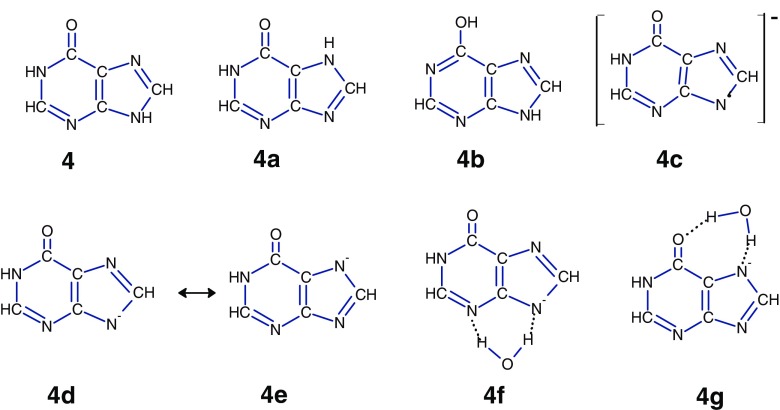
Most stable tautomers of hypoxanthine (**4**, **4a**, and **4b**), and the resonance structures for deprotonated hypoxanthine (**4d** and **4e**) and their hydrated complexes (**4f** and **4 g**

Theoretical and experimental studies [38, 70–72] indicate that in the gas phase hypoxanthine can exist mainly in two keto tautomeric forms, **4** and **4a**, (Scheme 5). The canonical structure **4** is calculated to be less stable than the **4a** by 3.5 kJ/mol; the next most stable tautomer, **4b**, is 22.6 kJ/mol higher in energy than **4a** [38].

The calculations [70] show that the **4a** tautomer represents about 80% of the population in the gas phase. The predicted concentration for **4b** would be less than 0.1%. Hydration shifts in the tautomeric equilibria toward the **4** form; in the case of the dihydrated species, the populations of the **4** and **4a** tautomers would be about 50% [70]. Also, quantum chemical and Monte-Carlo calculations [73] indicate that both species might be coexisting under similar tautomeric populations in neutral hypoxanthine aqueous solution. The resonance Raman spectroscopy and quantum chemical calculations study [74] reported that in solution the hypoxanthine anion is formed only via deprotonation of the N7H and N9H sites. Thus, based on these results, one may assume that [**H-**H]^–^ formed from **4** and **4a** by ESI, either in solution or within the droplets, represent a mixture of the deprotonated tautomers of similar populations. The negative Mulliken charge distribution predicted by the calculations [74] for the N3, N7, N9, and O10 atoms of the **4c** anion are equal to 0.509, 0.543, 0.541, and 0.589e, respectively. The charges at the N3 and N9 atoms are comparable with those of N7 and O10, and both these positions could be the reactive sites for water interaction with resonance structures **4d** and **4e** leading to the complexes **4f** and **4g**, as schematically depicted in Scheme 5. The acidity values calculated [38] for the most acidic sites, N9H in **4** (1383.8 kJ/mol) and N7H in **4a** (1386.2 kJ/mol), are consistent with the measured value (1389.1 ± 8 kJ/mol).

### Correlation Between Water Binding Energies and Acidities

**Figure 3:**
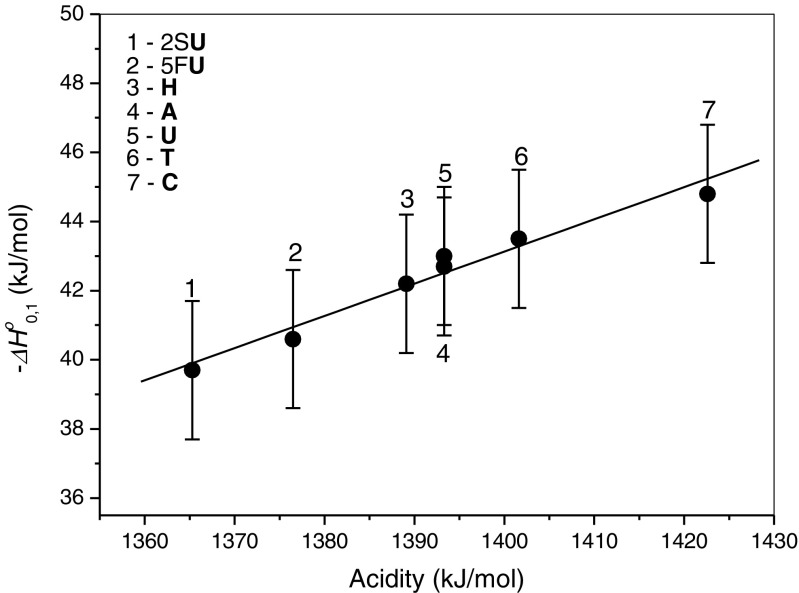
Plot of the binding energies, –*ΔH* ^*o*^_0.1_ , versus corresponding acidity of the most acidic site of nucleobases. The acidity values are given in Table 1

**Figure 4:**
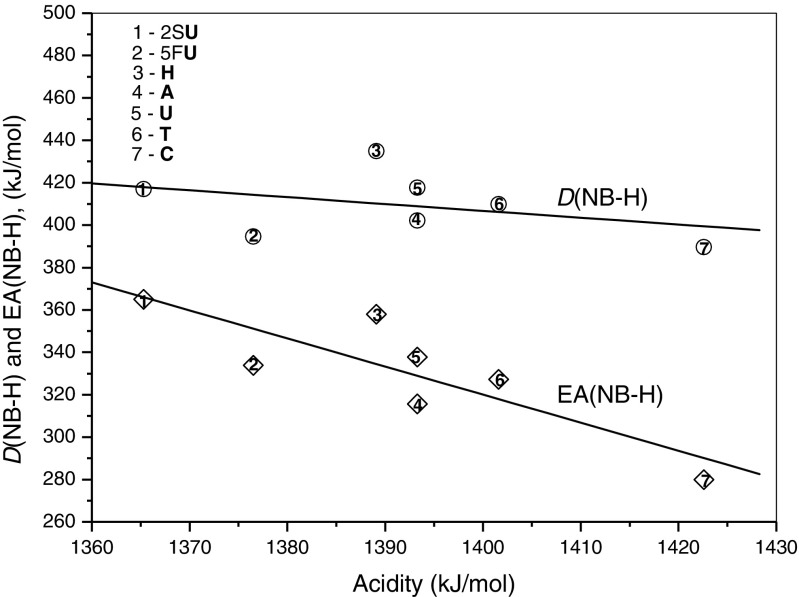
Plot of the N–H bond dissociation energies of **NB**, *D*(**NB-**H), and the electron electron affinities of the (**NB**-H) radical, EA(**NB**-H), versus corresponding acidity of the most acidic site of nucleobases. The acidity values are given in Table 1. The *D*(**NB**-H) and EA(**NB-**H) values for 5F**U**, **A**,**U**,**T**, and **C** are taken from Ref. [37]; for **H**, Ref. [14]. For 2S**U**, the EA(**NB-**H)=365 kJ/mol was estimated as the difference of *D*(NB-H) – E(DEA) based on E(DEA)=52.9 kJ/mol [12] and using for *D*(NB-H) the same value as for **U**

A plot of the binding energies (–*ΔH*^*o*^) of water molecule in the [NB-H]^–^٠(H_2_O) complexes versus the corresponding gas phase acidities of the most acidic site of NB is shown in Figure 3. The gas-phase acidity values used for this figure and also quoted in Table 1, except for 2S**U** [35], are obtained experimentally and reported in the literature [20, 31, 37–39]. A fair linear relation is observed in Figure 3. The correlation coefficient is 0.98. Changes in hydration enthalpies of [NB-H]^–^ can be thermochemically analyzed on the basis of the gas-phase acidity enthalpy, *ΔH*^*o*^_*ac*_, for deprotonation given by Equation (6)

6 $$ \varDelta {H}^o{{}_{ac}}_{=}D\left(\mathrm{N}\mathrm{B}\hbox{-} \mathrm{H}\right)\ \hbox{--}\ \mathrm{E}\mathrm{A}\left(\mathrm{N}\mathrm{B}\hbox{-} \mathrm{H}\right) + \mathrm{IE}\left(\mathrm{H}\right) $$

where *D*(NB–H) represents the bond energy for N–H broken during deprotonation of NB, EA(NB–H) the electron affinity of the [NB-H] radical, and IE(H) the ionization energy of the H atom. Since the IE(H) is constant, the *ΔH*^*o*^_*ac*_ should be dependent on the *D*(NB-H) – EA(NB-H) difference, which is related to the energy for dissociative thermal electron attachment by E(DEA) = *D*(NB-H) – EA(NB-H). For the systems studied, the approximate correlations in Figure 4 show that the EA(NB–H) values undergo larger change than those of *D*(NB-H). The slopes ratio of EA(NB-H)/*D*(NB-H) is equal to about 4. This implies that the electron affinity of the [NB-H] radical is the major factor determining the magnitude of the binding energy of water in the [NB-H]^–^٠(H_2_O) complexes, which is largely due to electrostatic attraction.

### Comparison to Neutral and Protonated Nucleobase

**Figure 5:**
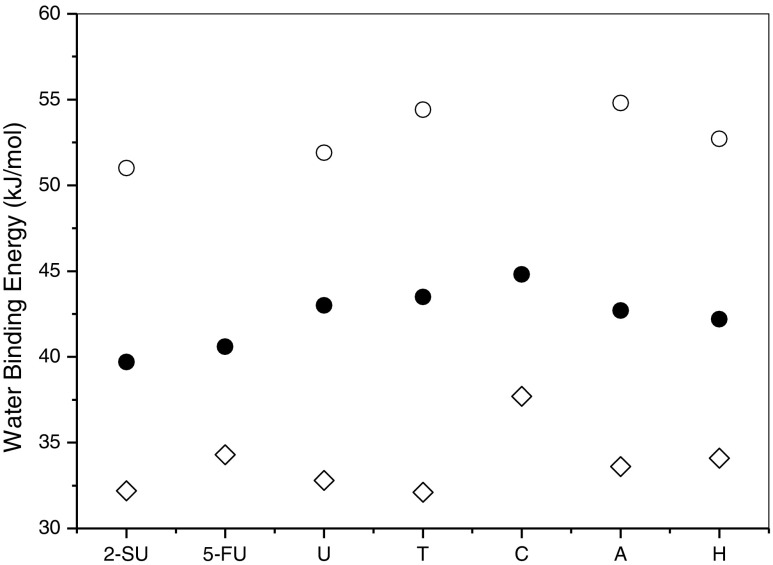
Comparison of the water binding energies for the neutral, NB⋅(H_2_O), (⋄); deprotonated, [NB – H]^−^⋅(H_2_O), (•); and protonated , [NB+H]^+^⋅(H_2_O), (ο), complexes. The binding energy values are given in Table 2

The hydration enthalpies obtained in this work for [NB-H]^–^٠(H_2_O) along with the literature values calculated [19, 26, 67, 72] for the neutral, [NB]٠(H_2_O), and those measured [45] previously for protonated forms, [NB+H]^**+**^٠(H_2_O), using the same methods employed here are compared in Table 2 and Figure 5. For all anionic complexes, the water binding energies are larger than those for the corresponding neutral complexes. This confirms the electrostatic nature of water interaction with the anionic forms of nucleobases. The stronger H-bonding interactions in the cationic complexes than those in anionic can be attributed to higher positive charge density concentrated on the site of [NB+H]^**+**^ protonation compared with a delocalized negative charge in the anionic nucleobases. For example, in the N1^–^ anions of [**U**-H] ^–^ and [2S**U**-H] ^–^, the large negative charge is located on the O2(S2) and O4 atoms [22, 42]. In [**A**-H] ^–^ and [**H**-H] ^–^, as discussed above, the negative charge is uniformly distributed on the N atoms. The electrostatic potentials calculated [76] for the deprotonated **A**, C, and **T** indicate that the negative charge is “spread” throughout the [**A**-H]^–^ anion, whereas in [**C**-H]^–^ the most of the negative charge resides in the C2=O region. The [**T**-H]^–^electrostatic potential is less delocalized than [**A**-H]^–^, but more than [**C**-H]^–^.

**Table 2:** Binding Energies (kJ/mol) of Water for the Monohydrated

NB	[NB]⋅(H_2_O)	[NB – H]¯⋅(H_2_O)	[NB+H]^+^⋅(H_2_O)
2S**U**	32.2^a,d^	39.7	51.0
5F**U**	34.3^a,e^	40.6	
**U**	32.7^a,f^	43.0	51.9
**T**	32.2^a,f^	43.5	54.4
**C**	37.2^a,f^	44.8	
**A**	33.6^b,f^	42.7	54.8
**H**	34.1^c,g^	42.2	52.7

Complexes of Neutral, Deprotonated, and Protonated Nucleobases

^a^ In the [NB]⋅(H_2_O) complexes, the water molecule is attached to the N1H bond and

the O2(or S2) atom of NB.

^b^ Complex formed between the N3 atom and the N9H

bond of the canonical tautomer.

^c^ Complex formed between the N1H bond and the

O10 atom of the keto-N9H tautomer.

The binding energies taken from:

^d^ Ref. [75].

^e^ Ref. [26].

^f^ Ref.[19].

^g^ Ref. [70].

The values for [NB–H]¯⋅(H_2_O), present work.

The values for [NB+H]^+^⋅(H_2_O), Ref. [45].

## Conclusions

In the present work, we have investigated the monohydration of deprotonated nucleobases produced by electrospray from alkaline solutions (pH ~10.5). The results from these experiments suggest that the pyrimidine nucleobases deprotonated at the N1 site are the dominant precursors for the hydrated complexes, [NB-H]^–^٠(H_2_O). In these systems, the water is most likely involved in a bidentate interaction with deprotonated nitrogen atom N1^–^ and the O2 (or S2) atom of the adjacent group. The measured hydration enthalpies for [**U**-H]^–^, [**T**-H]^–^, and [5F**U**-H]^–^ are very similar to the binding strengths calculated [30] for the corresponding hydrated complexes with water at the O2(N3) binding position. In the case of adenine and hypoxanthine, the [**A**-H]^–^ and [**H**-H]^–^ anions formed by deprotonation of the N9H and N7H tautomers are the precursors for the hydrated complexes, which for both systems most likely represent the mixtures of isomeric structures. The thermochemical properties found for the hydration reactions of [NB-H]^–^ are similar within experimental uncertainty. A correlation between the hydration enthalpies and the corresponding acidities of the most acidic site of nucleobases is observed.
